# Measuring and decomposing the inequality of maternal health services utilization in Western Rural China

**DOI:** 10.1186/1472-6963-14-102

**Published:** 2014-03-03

**Authors:** Xiaoning Liu, Wenlong Gao, Hong Yan

**Affiliations:** 1 Department of Epidemiology and Biostatistics, School of Public Health, Xi’an Jiaotong University College of Medicine, P.O Box 46, No.76 West Yanta Road, Xi’an, Shaanxi 710061, China; 2 Institution of Epidemiology and Health Statistics, School of Public Health, Lanzhou University, Lanzhou, Gansu 730000, China

**Keywords:** Maternal health care utilization, Rural Western China, Socioeconomic inequality, Decomposition

## Abstract

**Background:**

To measure socioeconomic inequalities in maternal health services in rural western China and to analyze the determinants’ contributions of inequalities.

Study design: a cross-sectional study.

**Methods:**

The data utilized in this study were obtained from a cross-sectional study from 10 provinces in rural Western China in 2005. Wealth index of household socioeconomic status was developed by using principle component analysis. Concentration index, concentration curve and decomposition of the concentration index were employed to measure socioeconomic inequality in maternal health services utilization.

**Results:**

For more than four times prenatal visits, the concentration index was 0.0605 (95% CI: 0.0603, 0.0607). The concentration index of hospital delivery was 0.0230 (95% CI: 0.0210, 0.0240) and the concentration index of more than 2 times postnatal visits was 0.0842 (95% CI: 0.0836, 0.0847). Han ethnicity woman, particularly in conjunction with high school education and rich wealth status, was the main contributor to inequality in maternal health services utilization.

**Conclusions:**

There is a strong pro-rich inequality of maternal health services in rural western China. This study suggests that an effective way to reduce the inequality is not only to narrow the gap of income between the rich and poor, but focus education on ethnic minority woman in rural remote areas.

## Background

Health inequality is used to designate differences, variations, and disparities in the health achievements of individuals and groups [1]. Researchers worldwide have focused on health inequality and population health. A study carried out among nine western industrialized countries exhibited a strong negative correlation existing between income inequality and life expectancy [2]. Some studies found a positive association existing between income inequality and mortality [1,3-5]. Meanwhile, some other studies also showed possible effects of income inequality on self rated health [6] and children’s malnutrition [7,8]. These findings suggested that income inequality is bad for the health of the whole population [1]. In contrast, access to health services is concentrated among those at the upper end of the socioeconomic spectrum, while socioeconomic status contributed to greater inequality of health services utilization [9-11].

China has achieved much in healthcare with its rapid economic development. But great inequality in population health exists due to its geographic and economic factors. For example, in 2005, the maternal mortality rate (MMR) was less than 20 per 100,000 live births in eastern coastal regions, which was approximately 4–5 times lower than that of in western regions. Meanwhile, the proportion of hospital delivery had reached nearly 100% at the county level of eastern coastal regions, but was 70% or less at the county level in western areas [12]. The contrasts of medical services in quality and accessibility between China’s large cities and its less developed rural areas were obvious. According to the Ministry of Health, numbers of doctors, hospital beds, the types of health care and medical treatments in large cites are approaching levels in industrialized countries. However, the number of village doctors decreased from 1.8 million to 0.8 million, as well as the number of health workers decreased from 3.4 million to 0.8 million during the same period [13].

Our previous study has described the utilization of maternal healthcare services, and analyzed the associated factors and assessed their regional differences among 10 provinces in rural western China. The purpose of this present study was to analyze the degree of income-related inequality of maternal health services utilization and to decompose socioeconomic inequality into its determinants. These findings can be used to make implications for the Chinese government to promote maternal healthcare utilization for rural resident in rural western China.

## Methods

The data utilized in this study were obtained from a cross-sectional study from 10 provinces in rural Western China in 2005. The background of this study and the selection of sites were described in our previous published paper [14].

Three outcomes were treated as maternal health services utilization index: (1) if a woman had more than four times prenatal visits; (2) if a woman had a hospital delivery; and (3) if a woman had more than two times postnatal visits. Determinants associated with maternal health services utilization were: woman’s age which are categorized into four levels (1: less than 20 years; 2: 20–29 years; 3: 30–39 years; 4: over 39 years), ethnicity (Han vs. minority), woman’s education (primary, secondary and high school), husband’s education (primary, secondary and high school), household wealth index (poor, middle, rich), and parity (1, 2, and more than 2).

The household wealth index is a commonly used measurement of socio-economic status of households. It was developed by using the first principle component analysis. The index combined information on a set of household asserts and living conditions: the resource of household income, the ownership of a television, bicycle and motorcycle, and the availability of clean water. The index was categorized into 3 socio-economic levels: poor, middle and rich households.

The concentration index (CI) is employed in this paper to measure maternal health services utilization inequality. CI quantifies the degree of income-related inequality in a health variable, and is becoming a standard tool for the measurement of income-related health inequality. It can be computed as twice the covariance of the health variable and a person’s relative rank in terms of economic status, divided by the variable mean according to Equation. The value of the CI can vary between −1 to +1. A negative values implies that a variable is concentrated among poorer people while the opposite is true for a positive value. When there is no inequality, the CI will be zero. The value of CI measures the severity of socio-economic inequality. The larger the absolute value of CI, the greater the disparity [15].

(1) $$C=\frac{2}{\mu }\mathrm{cov}\left(y_{i},R_{i}\right)$$

Where C is concentration index; *y*_*i*_ is maternal health services utilization index; *R*_*i*_ is the fractional rank of individual *i* in the distribution of socio-economic position; μ is the mean of the maternal health services utilization variable of the sample and cov denotes the covariance.

The concentration curve plots the cumulative percentage of the health variable (y-axis) against the cumulative percentage of the population, ranked by socio-economic status (x-axis). If, by contrast, the maternal health services utilization sector variable takes higher (lower) values among poorer people, the concentration curve will lie above (below) the line of equality. The farther the curve is under the line of equality, the more concentrated the maternal health services utilization variable is among the poor.

The CI of maternal health services utilization measures the degree of inequality, which can be decomposed into the contribution of various explanatory factors. According to this method, the CI of maternal health services utilization can be decomposed into contributions of determinants to income-related inequality using the method of decomposition of CI. Based on the linear additive relationship between the maternal health service utilization variable *y*_*i*_ the intercept α the relative contributions of *x*_*ki*_ determinants and the residual error *ϵ*_*i*_ in Equation 2, the concentration index can be rewritten as in Equation 3:

(2) $$y_{i}=\alpha +\sum_{k}\beta_{k}x_{\mathrm{ki}}+\epsilon_{i}$$

(3) $$C=\sum_{k}\left(\frac{\beta_{k}{\bar{x}}_{k}}{\mu }\right)C_{k}+\frac{GC_{\epsilon }}{\mu }$$

Equation 3 shows that the overall inequality in maternal health utilization has two components, a deterministic or “explained” component and an “unexplained” component. In the former component *β*_*k*_ is the coefficient from a regression of maternal health services utilization variable on determinant *k*, ${\bar{x}}_{k}$ is the mean of determinant *k*, μ is the mean of the maternal health service utilization index, and *C*_*k*_ is the CI for determinant *k*. In the latter component, *GC*_*ϵ*_ is the generalized CI for the error term.

The decomposition method was used with a linear, additively separable model. However, the maternal health service utilization variables in this study are non-linear. Probit regression models are employed to analyze the influences of determinants on the probability of maternal health services utilization. One possibility when deal with a discrete change from 0 to 1 is to use marginal or partial effects, which shows the change in an explanatory variable [11,16,17].

Data was analyzed using STATA 12 statistical software and MS Excel.

## Results

Table 1 shows the summary statistics for all the variables. 70.47% of the women in this study were 20–30 years old, and 63.3% of them were Han ethnicity. About 64% of the women completed secondary school, and 78% of the women’s husband completed secondary school. 58.16% of the women had only one child. Each socio-economic position had about 33% of households in surveyed women.

**Table 1:** Socio-economic characteristics of the surveyed women

	**Frequency (%)**
Women’s age (years)	
< 20	341 (2.46)
20-	9760 (70.47)
30-	3585 (25.88)
40-	164 (1.18)
Ethnicity(Han)	9003 (63.8)
Women education (years)	
Primary school	4951 (35.59)
Second school	4898 (35.20)
High school	4064 (29.21)
Husband education (years)	
Primary school	3033 (21.76)
Second school	5359 (38.45)
High school	5546 (39.79)
Parity	
1	8207 (58.16)
2	5094 (36.10)
≥ 3	810 (5.74)
Wealth	
Poor	4634 (32.95)
Middle	4822 (34.29)
Rich	4608 (32.76)

**Figure 1:**
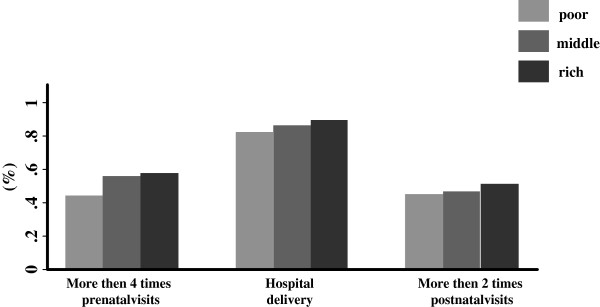
Wealth quintiles and maternal health services utilization.

In 2005, the rate of women had prenatal visits more than four times was 52.9%, the rate of hospital delivery was 86.2% and the rate of women had postnatal visits more than two times was 44.8%. These three outcomes increased with increasing socio-economic status. Only 44.3% women in poor location had prenatal visits more than four times which was significantly lower than that in middle and rich position (*χ*^2^ = 180.43, *p* <0.01). 81.88% of the poor women had hospital delivery compared to 89.6% among the rich position (*χ*^2^ = 115.05, *p* <0.01). 44.63% women in poor location had postnatal visits more than 2 times compared to 49.6% among the rich position (*χ*^2^ = 18.95, *p* < 0.01) (Figure 1).

**Figure 2:**
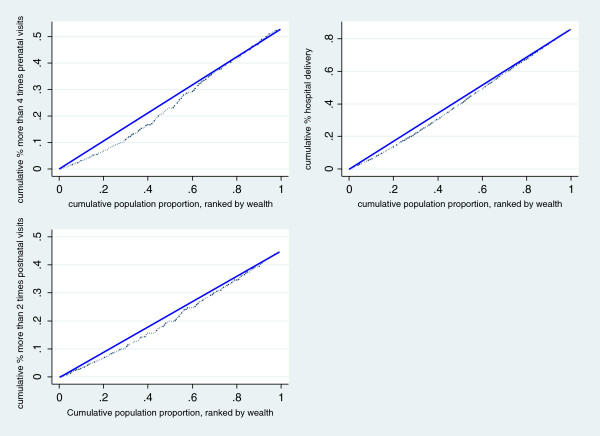
Concentration curve for maternal health services utilization.

The trend of inequality in maternal health services utilization for all three indicators was observed. The better the socioeconomic status was, the higher the maternal health services were utilized. The concentration index analyses further strengthened the above observation of inequality in maternal health services utilization for all three indicators. For the indicator of more than four times prenatal visits, the concentration index was 0.0605 (95% CI: 0.0603, 0.0607). The concentration index of hospital delivery was 0.0230 (95% CI: 0.0210, 0.0240) and the concentration index of more than 2 times postnatal visits was 0.0842 (95% CI: 0.0836, 0.0847) indicating a pro-rich inequality. From Figure 2 we can see that the concentration curve was under the line of equality, which indicated that these three outcomes are concentrated amongst the rich.

The decomposition analysis clarifies how each determinant contributes to the socioeconomic related inequality in maternal health services utilization. The contribution of each determinant depends on: 1) its impact on maternal health services utilization; and 2) how unequally distributed over wealth the determinant is. The results of the decomposition analysis are depicted in Table 2. The positive marginal effect indicates that the determinant had a positive association with outcomes and had high probability of outcomes compared to reference. The absolute value of contribution signifies the extent to which inequality attributed to this variable. The positive value of contribution means that the variable contributes to pro-rich inequality, that is to say, the richer individuals use more maternal health service than the poor, and vice versa. A determinant that has a high impact but with no wealth-related gradient will have less contribution to the overall concentration index as opposed to one with a high impact and high wealth-related inequality. As the variables were divided into categories, the contribution of each variable was generated by adding up contributions of variables within each category.

**Table 2:** Decomposition of inequality in the maternal health services utilization

	**More than 4 times prenatal visits**		**Hospital delivery**		**More than 2 times postnatal visits**	
	**Marginal effects**	**Contributions**	**Marginal effects**	**Contributions**	**Marginal effects**	**Contributions**
Women’s age						
<20	-					
20-	0.4015*	0.0226	0.1723*	0.0036	0.2247*	−0.0032
30-	0.5959*	0.0509	0.2657*	0.0069	0.1849*	0.0019
40-	0.4848*	0.0078	0.3139	0.0129	0.0592	0.0000
Ethnicity	0.3208*	0.0281	0.8199*	0.0158	0.1917*	0.0038
Parity						
1	-					
2	−0.2116*	−0.0153	−0.1746*	−0.0042	0.0147	0.0003
> = 3	−0.4654*	0.0041	−0.4451*	−0.0062	−0.0573	0.0063
Women education						
Primary school	-					
Second school	0.1848*	0.0066	0.2208*	0.0025	−0.0679*	0.0019
High school	0.3611*	0.0168	0.4035*	0.0058	−0.0377	0.0001
Husband education						
Primary school	-					
Second school	0.1476*	0.0077	0.0979*	0.0184	0.1115*	−0.0016
High school	0.2303*	0.0115	0.1869*	0.0041	0.1964*	−0.0025
Wealth index						
Poor	-					
Middle	0.2506*	0.0165	0.1887*	0.0023	0.0552*	0.0002
Good	0.2952*	0.0170	0.3590*	0.0037	−0.0479*	0.0007

*The marginal effects demonstrate associations between determinants and maternal health services utilization outcomes. Those with positive signs indicate positive associations with the probability of maternal health services utilization, while those with negative signs indicate negative associations. In addition, the larger the absolute value of a marginal effect, more substantial is the association. Statistically significant estimates of marginal effects are highlighted (p<0.05).

From Table 2, it is clearly observed that most of the inequality in maternal health service utilization can be explained by inequalities in women’s age, ethnicity, women’s education, husband’s education, and wealth. The variable parity seems to have an inequality reducing effect. Among these contributions, ethnicity, education and wealth made the greatest contribution to the inequality of maternal health service utilization. All of the contributions were positive, indicating that most of the pro-rich inequalities are affected by ethnicity, education and wealth. Therefore, woman with Han ethnicity and high school education had an above average probability of maternal health services utilization, were disproportionately concentrated in rich socioeconomic status groups.

**Figure 3:**
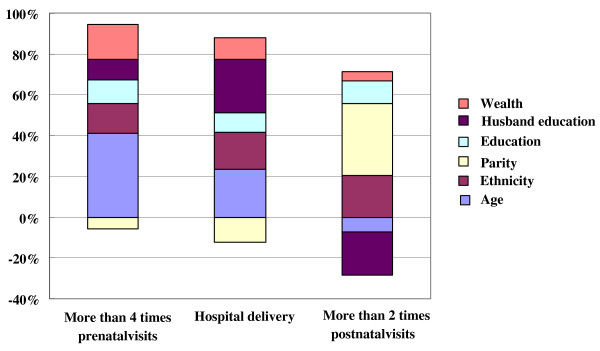
Decomposition of inequality in maternal health services utilization.

Figure 3 presents a summary of the contribution of the determinants to the overall concentration index of the variable. It is clearly observed that most of the inequality in maternal health services utilization can be explained by inequality in woman’s age, ethnicity, parity, education, husband’s education and wealth. But the effects of contribution of each variable to three outcomes were different.

## Discussion

This study focused on income-related inequality of maternal health services utilization and decomposed socioeconomic inequality into its determinants in western rural China. Most areas of rural western China are mountainous with underdeveloped economic conditions and poor health services, our previous study showed that the maternal healthcare were underutilized till 2005, and a great disparity existed in western rural China [14]. This study showed that the inequality existed in maternal health services utilization and socio-economic factor was the major factor accounted for the inequality. The three concentration index of maternal health services utilization were positive showed that obvious pro-rich inequalities of maternal health services utilization existed, that is, woman with a lower socioeconomic status are less likely to use maternal healthcare services, which indicates that a disproportionate share of maternal health services resources is utilized by richer people in spite of lower need. The results were similar to many others in health services studies in different parts of the world [10,18-20].

Compared to more than four times prenatal visits and more than two times postnatal visits, inequality in hospital delivery was lower. Since 2000, the China government and Ministry of Health have made continuous efforts and have issued a series of legislation to promote maternal health services and improve the inequality in maternal health services utilization in less-developed western rural areas. The comprehensive strategies included restrict on hospital delivery costs, provide appropriate subsidies for poor pregnant women, and strengthen the maternal health service quality in township hospital to improve the hospital delivery in rural western China [21]. These measurements could increase the rate of hospital delivery in poor women.

After decomposing the inequality of maternal health services utilization, this study showed that Han ethnicity woman, particularly in conjunction with high school education and rich wealth status, was the main contributor to inequality in maternal health services utilization. The result was consistent with previous studies that better wealth status was associated with increased maternal health services utilization. Better wealth status directly affects whether a woman can actually reach a facility for maternal health service and afford transportation costs [22,23]. Ethnicity and religion are often considered as markers of cultural background and are expected to influence beliefs, norms and values in relation to childbirth, service use and women’s status. Some studies showed that levels of maternal health services utilization were very low among ethnic minority women in rural China because of their traditional customs, such as a preference for home delivery [24-26]. Maternal education is consistently and strongly associated with all types of health behaviors. Education can increase knowledge of the benefits of preventive health care and awareness of health services, improve the ability of individuals to produce health by influencing their life style, and increase the use of health care services through improved knowledge, attitude and practice [23,27]. Some studies also found that education of woman is the major determinant of inequalities in maternal health services utilization [10,18]. In some African areas, women with higher education and lower socioeconomic status used more maternal health services than that of lower education and higher socioeconomic status [28].

At present, China’s strategy has largely focused on supply-side interventions through insured and subsidized delivery of care, and the improved quality of that care. It is necessary to explore a practical, sustainable development and low-cost maternal and children’s health model for western rural areas. Hence, bridging inequalities in the maternal levels of education is an important undertaking to narrow inequalities in the use of maternal health services. The government should focus education on rural woman, especially ethnic minority woman in rural remote areas in western China.

Beyond that, these three major contributors interact in different ways to determine use of health services. Low wealth status is indicated by low education and low income. In many societies, ethnicity and religion are closely linked to socioeconomic position and place of residence. Income and education are definitely linked to access, experiences and benefits from health care, which itself is a social determinant of health [29,30]. Furthermore, the results also show that residual variable contributed a lot to the inequality, suggesting that there remains a good deal of unexplained variation in inequality besides the variables examined in this analysis. It is important to note that redressing wealth inequalities alone can not be an effective intervention to inequalities in access to maternity care in the absence of intervention that also tackle the other social determinants such as education.

There are limitations to the study. First, fewer variables were used to compose the wealth index, which is used to measure the woman’s socioeconomic status, making it might be a poor representation of the woman’s actual economic situation. Second, there was likely to be recall bias because the subjects were interviewed with events that occurred a few years ago.

## Conclusion

There is a strong pro-rich inequality of maternal health services in rural western China. The inequality of hospital delivery was lower than that of more than four times prenatal visits and more than two times postnatal visits. As ethnicity, education and wealth index were the main factors contributing to the pro-rich inequality of maternal health services utilization. This study suggests that an effective way to reduce the inequality is not only to narrow the gap of income between the rich and poor, but focus education on ethnic minority woman in rural remote areas as well.

## Competing interests

The authors declare that they have no competing interests.

## Authors’ contributions

XL designed the prescription study, collected the data, conducted the data analysis and prepared the manuscript; HY contributed to the design and analysis of the study and the preparation of the manuscript; and WG assisted with the data analysis and reviewed the manuscript. All authors read and approved the final manuscript.

## Pre-publication history

The pre-publication history for this paper can be accessed here:

http://www.biomedcentral.com/1472-6963/14/102/prepub
